# Effects of Maternal Cigarette Smoking on Trace Element Levels and Steroidogenesis in the Maternal–Placental–Fetal Unit

**DOI:** 10.3390/toxics11080714

**Published:** 2023-08-19

**Authors:** Martina Piasek, Lana Škrgatić, Antonija Sulimanec, Tatjana Orct, Ankica Sekovanić, Jelena Kovačić, Anja Katić, Karmen Branović Čakanić, Alica Pizent, Nataša Brajenović, Andreja Jurič, Irena Brčić Karačonji, Zorana Kljaković-Gašpić, Blanka Tariba Lovaković, Maja Lazarus, Sandra Stasenko, Iva Miškulin, Jasna Jurasović

**Affiliations:** 1 Institute for Medical Research and Occupational Health, Ksaverska cesta 2, 10000 Zagreb, Croatia; mpiasek@imi.hr (M.P.); asulimanec@imi.hr (A.S.); torct@imi.hr (T.O.); asekovanic@imi.hr (A.S.); jkovacic@imi.hr (J.K.); akatic@imi.hr (A.K.); apizent@imi.hr (A.P.); nbrajen@imi.hr (N.B.); ajuric@imi.hr (A.J.); ibrcic@imi.hr (I.B.K.); zorana@imi.hr (Z.K.-G.); btariba@imi.hr (B.T.L.); mlazarus@imi.hr (M.L.); 2 University Hospital Centre, Petrova 13, 10000 Zagreb, Croatia; lana.skrgatic@mef.hr (L.Š.); iva.miskulin@gmail.com (I.M.); 3 School of Medicine, University of Zagreb, Šalata 3, 10000 Zagreb, Croatia; 4 Croatian Veterinary Institute, Savska cesta 143, 10000 Zagreb, Croatia; branovic@veinst.hr; 5 Merkur University Hospital, Zajčeva ulica 19, 10000 Zagreb, Croatia; sandra.stasenko@gmail.com

**Keywords:** cadmium, estradiol, lead, progesterone, umbilical cord sex steroids

## Abstract

This study evaluates the interaction of toxic elements cadmium (Cd) and lead (Pb) due to exposure from cigarette smoking, essential elements, and steroidogenesis in the maternal–placental–fetal unit. In a cohort of 155 healthy, postpartum women with vaginal term deliveries in clinical hospitals in Zagreb, Croatia, samples of maternal blood/serum and urine, placental tissue, and umbilical cord blood/serum were collected at childbirth. The biomarkers determined were concentrations of Cd, Pb, iron (Fe), zinc (Zn), copper (Cu), and selenium (Se), and steroid hormones progesterone and estradiol in maternal and umbilical cord blood and the placenta. Three study groups were designated based on self-reported data on cigarette smoking habits and confirmed by urine cotinine levels: never smokers (*n* = 71), former smokers (*n* = 48), and active smokers (*n* = 36). Metal(loid)s, steroid hormones, urine cotinine, and creatinine levels were analyzed by ICP–MS, ELISA, GC–MS, and spectrophotometry. Cigarette smoking during pregnancy was associated with increased Cd levels in maternal, placental, and fetal compartments, Pb in the placenta, and with decreased Fe in the placenta. In active smokers, decreased progesterone and estradiol concentrations in cord blood serum were found, while sex steroid hormones did not change in either maternal serum or placenta. This study provides further evidence regarding toxic and essential metal(loid) interactions during prenatal life, and new data on sex steroid disruption in cord serum related to cigarette smoking. The results indicate that umbilical cord sex steroid levels may be a putative early marker of developmental origins of the future burden of disease related to harmful prenatal exposure to cigarette smoke.

## 1. Introduction

The first physiological microenvironment of a human being is the maternal womb, which has an impact on long-term offspring health. Intrauterine life is a critical window of susceptibility during human development, as it is the most sensitive period when reactions occur to a complex interplay of intrinsic maternal–offspring biological processes as adaptive responses to extrinsic factors of the surrounding environment of the emerging individual phenotype, known as prenatal programming or fetal programming [1,2]. During prenatal life, maternal nutrient deficiencies and environmental stressors, such as exposure to environmental toxicants, can disrupt fetal growth, which may have a lifelong impact on individual health. Increased risks of developing health disorders and diseases in adulthood due to prenatal or perinatal conditions were recognized as early as 1934, and increasingly studied later on, including by British physician and epidemiologist David J. Barker and coworkers, who proposed the hypothesis on fetal origins of adult disease, which has now been largely accepted as paradigm of the developmental origins of health and disease (DOHaD) [2,3,4]. Epidemiological studies of infant and adult mortality have revealed associations between birth weight and infant mortality with metabolic, cardiovascular, and behavioral disorders in later life due to developmental plasticity during epigenome–environment interaction, which permanently shapes the morphology, function, and metabolism of the organism, therefore contributing to the adult health condition [2,5].

In recent decades, metal(loid)s have been a source of constantly growing concern due to their pervasive occurrence and rise in the human environment [6]. Cigarette smoking is an additional major source of daily exposure to cadmium (Cd) and lead (Pb), as tobacco smoke, a mixture of thousands of toxic, genotoxic, and mutagenic substances, also contains toxic metal(loid)s. In women of reproductive age, toxic metal(loid) exposure-caused adverse effects can extend to their progeny. Active maternal cigarette smoking before and during pregnancy may cause myriad adverse effects, including disruptions of placental functions and pregnancy outcome, as well as the health of the offspring from conception onward, including reproductive development [7,8]. The mechanisms of adverse subsequent health effects involve adaptive changes of the epigenome (DNA methylation, histone modifications, and expression of noncoding microRNA), an area of intensive study which has yet to fully elucidate just how it affects postnatal offspring health [9,10,11]. It is possible that the transgenerational epigenetic effects reported in mammals are caused by a number of environmental natural and synthetic xenoestrogens, including metal(loid)s, by virtue of their potential to act as endocrine disrupting chemicals (EDCs), as evidenced experimentally both in vivo and in vitro [12,13,14,15]. The maternal, placental, and fetal compartments (fetal liver and adrenal glands) involved in sex steroid biosynthesis, including progesterone (P4) and estradiol (E2), act as a unique, combined, functional organ called the maternal–placental–fetal unit, or feto–placental unit, which is crucial in normal development during intra- and extrauterine life [16,17,18,19,20]. Emerging work from the perspective of DOHaD suggests that the prenatal hormonal milieu may contribute to later health and disease risk, including neurodevelopmental and behavioral disorders, fertility impairments, contemporary chronic diseases, and malignancies, such as prostate and breast cancers [15,21].

Human biomonitoring (HBM) is an accepted epidemiological method for the assessment of actual exposure levels and health risks in studied population groups based on the fact that chemicals, including bioaccumulative metal(loid)s, leave a detectable trace in the organism during and/or after exposure [22,23,24,25]. Although blood is considered the most suitable sample to assess recent individual exposure, it is not an ideal matrix to trace bioaccumulated chemicals, as its sampling requires an invasive procedure that involves ethical and practical constraints, particularly in vulnerable population groups of children and pregnant women. Noninvasive sample use has therefore been increasingly encouraged for repeated or routine sampling during HBM as a cost-efficient and toxicologically relevant alternative [23,26]. Knowledge on the necessity for HBM of early life stages, including measurements of metal(loid)s in noninvasive biological specimens accessible only during the period of pregnancy and lactation, started to emerge some four decades ago [27,28]. Human environmental exposure to metal(loid)s and their retention in the body can be assessed postpartum and during lactation in umbilical cord blood, placenta, meconium, maternal and infant urine, breast milk, and hair samples, which also offer valuable information on their transfer between mother and child. With this regard, the human placenta, as the organ at the maternal–fetal interface, has been gradually recognized as an easily accessible tissue sample for assessing metal exposure and effects in expectant mothers and the fetus by analyses ex vivo, and in cultures of placental tissue in vitro [29,30,31,32]. We have been using placental tissue as a specimen in our research for more than two decades to measure levels of Cd, Pb, iron (Fe), zinc (Zn), and copper (Cu) [7,33,34,35,36,37]. Moreover, analysis of sex steroid hormones in maternal blood/serum, as well as in the placenta and umbilical cord blood/serum, should continue to be used in further studies of adverse reproductive and perinatal effects of toxic metal(loid)s (as reviewed in [7,13,14,34]).

A current focus in the research related to lifetime health risks due to hazardous intrauterine exposure to toxic metal(loid)s includes maternal and fetal steroidome disruptions [17] through changes in antioxidant regulation and the epigenome [37,38,39]. This study aimed to assess the interaction between prenatal exposure to the main toxic metals Cd and Pb from cigarette smoke and the levels of selected essential trace elements Fe, Zn, Cu, and selenium (Se), as well as and P4 and E2 in the maternal–placental–fetal unit, hypothesizing that these indices may serve as putative biomarkers of effects, and potential contributing factors of individual DOHaD.

## 2. Materials and Methods

### 2.1. Study Population

The study participants included 155 postpartum women (mother–infant pairs) recruited during 2018 and 2019 in two clinical hospitals in Zagreb, Croatia. The inclusion criteria for recruitment were healthy women with spontaneous vaginal delivery at term (37–42 gestational weeks), having no chronic diseases or major complications before and/or during pregnancy or at childbirth. Complying with the principles of the Helsinki Declaration, before enrolment, all participants were informed by medical staff in the maternity ward about the study goals, data, and planned sample collection, ensuring their anonymity by coding all personal data during the publishing of study results. Each study participant was also acquainted with the right to withdraw at any time during or after the study, and signed an informed consent form.

Personal and clinical data were collected from each participant and recorded in questionnaire form designed from experience and used in previous studies [33,36,40]. The recorded data collected from the study participants included: sociodemographic characteristics (age, education, dietary and smoking habits); potential sources of environmental and professional toxic metal exposure (place of residence and occupation); anthropometric characteristics (maternal height, body weight before pregnancy, and weight gain during pregnancy); obstetrics and gynecology history (number of previous abortions, miscarriages, and pregnancies); and potential health problems during pregnancy (increased blood pressure, blood glucose level, peripheral edema, or other major health problems, which also served as exclusion criteria). Clinical data on postpartum women and infants recorded by the obstetrician included: maternal weight at delivery, newborn sex, birth weight, birth length, and APGAR score in the 1st and 5th minute after birth.

Based on self-reported information on cigarette smoking habits, study participants were originally planned to be divided into two groups, as used in our previous studies [33,36,40], i.e., into ‘nonsmokers’—persons who have never smoked, or stopped smoking more than 12 months prior to the last pregnancy, and ‘smokers’—persons who smoked any time during the pregnancy or within the 12 months prior to pregnancy. However, after a closer look at the data related to the self-reported smoking habit, and maternal urine cotinine and Cd levels data analysis, study participants were instead divided into three study groups for further statistical analysis: ‘never smokers’, ‘former smokers’—persons who smoked > 100 cigarettes during their lifetime (according to [41]) and/or stopped smoking during the first trimester (considering that the placenta is fully developed by the end of the first trimester), and ‘active smokers’—persons who continued smoking during pregnancy.

### 2.2. Sample Collection

All samples of mother–infant pairs were collected according to the study protocols in the maternity ward, and transferred to the analytical laboratories of the Institute for Medical Research and Occupational Health for further preparation and analysis. Maternal urine was collected at admission, before delivery, whereas maternal peripheral venous blood, umbilical cord blood, and placenta were collected immediately after childbirth.

Urine samples were collected in 100 mL colorless PP containers with screw-cap (REF 75.562.005; Sarstedt, Nümbrecht, Germany), blood samples in two vacutainer trace element tubes (REF 368380, silica-coated for serum and REF 368381, with K2EDTA as an anticoagulant for blood; Beckton-Dickinson, New Jersey, NJ, USA), and whole placentas were placed in zip-lock polyethylene bags. All samples were received in the analytical laboratory within two hours of delivery, where they were aliquoted (urine), further prepared (serum separated and placental tissue sampled), and stored at −80 °C or −20 °C until analysis.

Plasma and serum samples were separated by centrifugation for 20 min at 3000 rpm (Hettich Rotanta/R, type 3501, Tuttlingen, Denmark), transferred into cryo-polypropylene tubes (REF 72.379; Sarstedt, Nümbrecht, Germany), and stored at −80 °C until analysis. Two aliquots of urine samples in 5 mL vials (REF 60.558; Sarstedt, Nümbrecht, Germany) and whole blood samples in collection tubes were stored at −20 °C.

Sampling of the placenta was based on previous experience and procedures described earlier [33,36,40]. The whole placenta was placed on the maternal side (basal plate) and blotted on absorbent material, the umbilical cord and extraembryonic membranes were trimmed, and fresh placental mass recorded. To ensure representative tissue samples, and prevent external metal contamination, each placenta was cut using a ceramic knife along the entire organ to obtain three full-thickness, large sample chunks of approximately 30 g each: one from the central region avoiding the umbilical cord insertion (part C), and two from between the central region and periphery, the outermost 3 cm within the placental disc edge (parts P1 and P2). From each larger part, smaller parts were taken, from which about 2 mm thick outer layers were cut off from both the fetal (chorionic membrane) and maternal side (decidua basalis) to obtain samples of mostly trophoblastic tissue weighing approximately 1–2 g fresh tissue weight for element analysis, and 0.5–1 g for steroid hormone assay. The samples were stored in 5 mL screw cap vials (REF 60.558; Sarstedt, Nümbrecht, Germany) at −80 °C until further preparation for each analysis.

### 2.3. Cotinine and Creatinine Analysis

Cotinine from maternal urine was extracted with an 85 µm polyacrylate fiber (Supelco, Bellefonte, PA, USA) using headspace–solid phase microextraction (HS–SPME) at 80 °C for 15 min, and determined by gas chromatography–mass spectrometry (GC–MS; Varian 3400 CX gas chromatograph equipped with a Saturn 4D ion trap mass spectrometer) [42]. The creatinine in maternal urine samples was measured spectrophotometrically (Cary 50, Varian, Australia) at 492 nm, using the Jaffe reaction.

### 2.4. Element Analysis

All preparation (sample preparation, digestion, and analysis) was performed in a laboratory equipped with a heating, ventilating, and air conditioning system combined with high-efficiency particulate air (HEPA) filters. Element concentrations in blood and placenta samples were determined by inductively coupled plasma–mass spectrometry (ICP–MS) Agilent 7500cx (Agilent Technologies, Tokyo, Japan).

Three samples (C, P1, and P2) of each placenta were digested with concentrated nitric acid and ultrapure water (1:1) in a microwave digestion system UltraCLAVE IV (Milestone, Sorisole, Italy), adjusted to 5 g with ultrapure water (GenPure, TKA System GmbH, Germany), and stored at 4 °C until element analysis. Before analysis, placenta samples were 20-fold diluted with a solution of 1% (*v*/*v*) HNO_3_ and 3 µg/L internal standards. The average concentration of element levels from all of the sections, the central (C) plus peripheral (average of P1 and P2) parts, were used for statistical analysis since no significant differences were detected between placental samples (C and pooled P1 and P2 samples, tested by *t*-test for dependent samples).

Blood samples were 70-fold diluted with an alkaline solution containing 0.7 mmol/L NH_3_, 0.01 mmol/L EDTA, 0.07% (*v*/*v*) TX-100, and 3 µg/L internal standards (germanium (Ge), rhodium (Rh), terbium (Tb), lutetium (Lu), and iridium (Ir)).

Urine samples were 20-fold diluted with a solution of 1% HNO_3_ and 3 µg/L internal standards (Ge, Rh, Tb, Lu, and Ir). All element concentrations in urine were expressed per gram of creatinine.

Commercially available reference materials for blood (SeronormTM Trace Element Blood (Levels I, II and II) and ClinChek^®^ Whole Blood Control (Levels I, II and III)), urine (SeronormTM Trace Elements Urine (Levels I and II) and ClinChek^®^ Urine Control for Trace Elements (Level I and II)), and tissue (pig kidney BCR 186R and bovine muscle ERM-BB 184 (IRMM, Geel, Belgium)) were used to check analytical accuracy. Overall recoveries were 90–108% of the assigned values. Additionally, the laboratory participates in a monthly, international, external quality assessment scheme for these metals in human blood, serum, and urine, and over the past two years, the obtained z-scores were < 2 for the elements in blood examined here.

### 2.5. Progesterone and Estradiol Analysis

Levels of P4 and E2 in maternal and cord serum and the placenta were analyzed by solid phase enzyme-linked immunosorbent assay (ELISA), based on the principle of competitive binding, using Demeditec kits for P4 (DE1561) and E2 (DE2693), and following the manufacturer’s instructions (Demeditec Diagnostics GmbH, Kiel, Germany).

Thawed samples were washed briefly 2 to 3 times in saline solution (0.9% NaCl), blotted on filter paper, weighed, and minced with metal scissors into small pieces following the addition of 75% ethanol to obtain a 10% *w*/*v* homogenate (approximately 2.5 mL EtOH). Samples were left overnight at 4 °C, and the next day homogenized on ice with an Omni tissue homogenizer (TH220-PCRH, OMNI International, PerkinElmer Inc., Kennesaw, GA, USA). Then, 500 µL homogenate was taken, and 3 mL 75% ethanol was added, vortexed, and 1.75 mL diluted homogenate pipetted into 2.0 mL tubes (Eppendorf, Hamburg, Germany) and centrifuged at 15,000× *g* for 15 min. Supernatants were decanted, freeze-dried (Hetosic, HETO Ltd., Birkerød, Denmark), and stored at −20 °C until steroid analysis.

Due to the high levels of P4 and E2 at delivery, serum samples were diluted to fall within the calibration curve range. According to the manufacturer recommendations, we used “Steroid free serum” (DE101561) for P4 and “Standard 0” (DE2693CAL0) solution for E2 analyses. Serum samples were diluted 20-fold for P4 in maternal serum, 40-fold for P4 in cord serum, and 20-fold for E2 in maternal and cord serum. Analysis of the samples that were outside the calibration range was repeated with 80-fold dilutions for P4 in cord serum (*n* = 14), and 40-fold serum dilutions for E2 in maternal serum (*n* = 4). Before ELISA analysis, placental supernatant lyophilizates were reconstituted with 1 mL saline solution, and then additionally diluted 50-fold with “Steroid free serum” for P4 and 3-fold with “Standard 0” for E2 analysis. A TECAN Infinite F50 plate reader (TECAN Group Ltd., Männedorf, Switzerland) was used to measure absorbance at 450 nm. The P4 and E2 concentrations were calculated using 4-parametric logistic regression methods, and their average concentrations from C and P placental sections were used for statistical analysis.

### 2.6. Statistical Analysis

All numerical variables (maternal age, body mass index (BMI), weight gain during pregnancy, parity, smoking habit, cotinine level, newborn characteristics, concentrations of Cd, Pb, and essential elements, levels of P4 and E2) are presented by median (min–max or 25–75% interquartile range, IQR) and/or mean with standard deviation (SD). Elements and cotinine concentrations below the limit of detection (LOD) were substituted with LOD/2 values. For categorical variables (education level, passive smoking, newborns’ sex), the number of observations per category is shown. Differences between the three groups (never smokers, former smokers, and active smokers) in numerical variables were tested by the Kruskal–Wallis test (at *p* < 0.05), and Dunn’s multiple comparisons test with Bonferroni adjustment was used as a post hoc test. When the comparison included only two groups (differences in smoking habits between former and active smokers), Mann–Whitney’s test was used. Fisher’s exact test was used to test differences between categorical variables. Spearman’s rank correlation was used to present the relationships between variables. For variables that were significantly associated with active smoking in univariate analyses, multiple linear regression analysis was performed. In addition to smoking status, age, education (university degree vs. primary or secondary school), passive smoking, prepregnancy BMI, weight gain during pregnancy, and parity were included as covariates in all models. Dependent variables were log-transformed to improve normality of residuals.

A statistically significant level was set at 5% (*p* < 0.05). Statistical analyses were performed using R, version 4.1.3 (R Foundation for Statistical Computing, Vienna, Austria) and TIBCO Statistica, version 13.5.0.17 (TIBCO Software Inc., Palo Alto, CA, USA).

## 3. Results

The main general characteristics of the study participants and their infants are presented in Table 1. The average age of all participants was 31.7 ± 4.7 years. Except for smoking, no other differences existed between the study groups with regard to other sources of the measured metals’ exposure, such as diet (in the self-report, all subjects reported having a mixed diet), ambient, or occupational environment. Compared to the groups of former and never smokers, active smokers were significantly younger and had lower education levels. There were no significant differences between the study groups in BMI before pregnancy, weight gain during pregnancy, or parity. Active smokers started smoking at a younger age compared to former smokers, while the duration of smoking and smoking index were comparable between these two study groups. Most of the former smokers (20 out of 48) stopped smoking between pregnancy weeks 4 and 6, i.e., at the time of confirmation of pregnancy, while 13 subjects stopped smoking within 15 months before the pregnancy, 13 stopped > 15 months (ranging from 21 months to 11 years) before the pregnancy, while two former smokers who reported no smoking during the pregnancy did not provide data when exactly they stopped smoking. Exposure to secondhand passive smoke was self-reported by about half of never smokers (55%) and former smokers (48%), while in active smokers, additional secondhand smoking was more often and reported by 69% participants. Active smokers had the highest urine cotinine levels, while never smokers and former smokers had median cotinine levels below 5 ng/mL, and concentrations < LOD were obtained in 41% of never smokers and 33% of former smokers. All newborns were of excellent health, with the highest APGAR scores of viability at birth in the first and fifth minutes being 10/10 or 9/10. Approximately 60% of newborns were males, and the difference in newborn gender between the study groups did not differ. Average birth weights and lengths reflected the apparent good health of the infants. There was a highly significant (r = 0.80; *p* < 0.001) positive correlation between birth weight and birth length. Newborns of active smokers had a tendency towards a lower birth weight, which was not significant (only one participant delivered male infant with low birth weight of 2390 g). An overall effect of maternal cigarette smoking was found for the ratio between birth weight and placental weight. No differences were found in birth weight, birth length, trimmed placental weight, and the ratio between birth weight and placental weight across the study groups stratified by newborn sex. When these parameters were tested between the participants designated as never smokers plus former smokers (*n* = 119) vs. active smokers (*n* = 36), in boys (*n* = 71 vs. *n* = 23 boys), borderline differences in birth weight (*p* < 0.052) and significant birth weight:placental weight ratios (*p* < 0.035) were found.

Average and median concentrations of Cd and Pb in maternal urine and blood, placenta, and cord blood related to maternal smoking habits are presented in Table 2. Maternal cigarette smoking significantly increased Cd levels in maternal blood, placenta, and cord blood. Median Pb concentrations in placenta were increased, while there were no significant differences in Pb levels of maternal urine, maternal blood, and cord blood between the study groups.

Average and median concentrations of the measured essential elements in maternal urine and blood, placenta, and cord blood related to maternal smoking habits are presented in Table 3. Placental Fe levels were lower in active smokers compared to Fe placental levels in either never smokers or former smokers. No differences were observed in Zn and Cu levels in maternal, placental, and cord blood in relation to maternal smoking habits. In active cigarette smokers, the median maternal urine Se levels were decreased compared to never smokers and former smokers.

**Figure 1:**
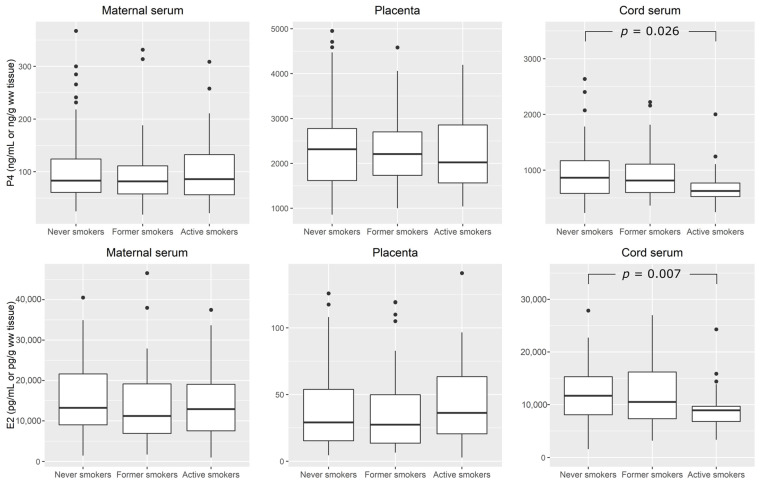
Progesterone (P4) and estradiol (E2) in mother–newborn pairs in relation to maternal smoking: never smoked (*n* = 71), former smokers (*n* = 48), and active cigarette smokers (*n* = 36). Differences between groups were tested by Kruskal–Wallis test (at *p* < 0.05), and Dunn’s multiple comparisons test with Bonferroni adjustment was used as a post hoc test.

Levels of P4 and E2 in mother–newborn pairs in relation to maternal smoking habits, irrespective of sex of newborn, are presented in Figure 1. There were no differences in either maternal serum P4 or placental P4 levels related to maternal smoking habits. Conversely, median (IQR) cord serum P4 was significantly lower in active smokers (626 (517–809) ng/mL) than in never smokers (865 (583–1169) ng/mL). No significant differences in E2 levels were found in maternal serum and placenta in relation to maternal smoking. Again, median (IQR) cord serum E2 in newborns of active smokers (8930 (6580–9701) pg/mL) were significantly lower than in never smokers (11,700 (8086–15,290) pg/mL).

Multiple regression analysis (Table 4) confirmed the association of active smoking with a decrease of P4 and E2 levels in cord serum. A decrease of cord serum P4 was also associated with increased parity and lower weight gain, while a decrease in cord serum E2 was associated with increased parity. Multiple regression analysis also confirmed effects of cigarette smoking on increased levels of Cd in all measured samples and Pb in placenta, as well as decreased Fe in placenta. However, the effect of cigarette smoking was not confirmed for decreased Se in maternal urine. Additionally, the participants’ age had effects on an increase of Cd in urine and placenta, lower education level on Cd in urine, and passive smoking on maternal blood Cd. Higher weight gain was associated with an increase in placental Pb levels. The effect of maternal cigarette smoking on the ratio between birth weight and placental weight was not confirmed by regression analysis.

Spearman correlation coefficients between P4 and E2 levels in the three measured samples (maternal serum, placenta, and cord serum) and newborn data are shown in Table 5. We found significant positive correlations between maternal serum P4 and both placental and birth weight, as well as between cord serum P4 and birth length, while placental E2 was negatively correlated with birth weight.

## 4. Discussion

We studied biomarkers that may serve as predictive adaptive responses of the fetus to maternal cigarette smoking as a specific environmental stressor in utero, and the consequent postnatal mismatch between prenatal and postnatal environments. The emphasis of the study was on the fetal origins of disorders in essential element levels during gestation (Fe, Zn, Cu, and Se) as potential pathways into metabolic disorders, such as obesity in childhood and adulthood, and the steroid hormones during pregnancy (P4 and E2 in maternal serum, placenta, and umbilical cord blood serum) to account for the observations based on the DOHaD paradigm due to environmental influences [5,43,44].

### 4.1. Trace Elements in Mother–Infant Pairs

Toxic metals from cigarette smoke in urine and in maternal and cord blood. Maternal self-reported smoking habit during pregnancy was confirmed by increased urinary cotinine levels that clearly differentiated never and former smokers from active smokers. Active smoking status validation by urinary cotinine analysis minimized potential biases associated with relying solely on self-reported smoking status. Such approach is consistent with the findings of other studies, which have emphasized the importance of validating smoking status to ensure accurate risk assessment [45]. We found increased Cd concentrations in both the mother and fetus, with higher maternal urinary Cd in active vs. never smokers, and higher maternal and cord blood Cd than in both former and never smokers. Median and IQR urinary concentrations of Cd in our participants were <1 µg/g creatinine, which can be considered a ‘normal’ or ‘reference’ concentration [46], and within the guidance values of the recently proposed urinary Cd ‘alert’ values according to age (approximately 0.23–0.63 µg/g creatinine for the age range in our study) [47]. Maternal median blood Cd values were somewhat lower than in our previously studied cohort [36], comparable to recent biomonitoring data for Cd blood concentration for the U.S. and European populations [48,49,50], and below the reference value of 1 µg/L for adults [51]. Urinary Cd increases with the Cd body burden, and age and is generally considered to reflect long-term Cd exposure. A positive association between participants’ age and urinary Cd concentration was also identified in our study group. On the other hand, Cd in blood reflects both long-term and recent exposure, and partially reflects the accumulated body burden over recent weeks and months [46]. After a decrease in exposure, the decrease of Cd in blood is more rapid than the decrease in body burden. Therefore, former smokers generally have higher urinary Cd than never smokers, but lower blood Cd levels than active smokers. In this study the same trend was also observed, although no significant difference was found in urinary Cd between never smokers and former smokers (*p* = 0.1), due in part to the relatively small number of study participants and the applied conservative statistical analysis (post hoc Dunn’s multiple comparisons test with Bonferroni adjustment). Smoking cessation resulted in an expected and rapid decrease in blood Cd, with a difference seen between former smokers and active smokers. Interestingly, we also found higher cord Cd in active smokers than in former smokers or never smokers, although the measured Cd levels in cord blood were expectedly very low. Even though the fraction of Cd passing the placenta is small, it appears to increase with maternal smoking, as we have also previously found and discussed [36].

Whole blood Pb concentrations have been used for decades to assess Pb exposure, including for biomonitoring purposes. Despite the noninvasive collection method, spot urinary Pb is considered unreliable because of the wide biological variations. Furthermore, blood Pb reflects a combination of recent and past exposure over several years, while urinary concentrations reflect Pb diffused from plasma and excreted through the kidneys [52,53]. We observed a further decrease in Pb levels in maternal and cord blood and placenta in the Croatian population compared to the levels reported in the cohort recruited about 10 years ago [36]. These results are in line with reported reduced exposure to Pb, largely due to a drop in air pollution, and steadily declining Pb biomarker concentrations in populations across Europe [54,55] and the U.S.A. [50]. Therefore, blood Pb levels were comparable with the results of European, U.S., and Canadian biomonitoring surveys performed in the last decade [48,49,50,56,57]. Maternal urinary Pb concentrations in the present study were somewhat higher than in females from the U.S. NHANES 2011–2016 studies [50], and lower than in pregnant women in the HEALS-EXHES study [57]. Our result suggests low Pb in utero exposure, even in smoking mothers, as in only 3% of umbilical cords were Pb levels somewhat higher than the level of concern for developmental neurotoxicity of 12 µg/L (95th percentile lower confidence limit of the benchmark dose of 1% extra risk, BMDL_01_) [58]. Although a weak but consistent trend in increasing Pb median concentrations can be noted in all measured biological samples from never, former, and active smokers, there were no effects of smoking on a statistically significant increase in Pb urine or blood levels.

Toxic metals from cigarette smoke in the placenta. The literature data on comparative levels of toxic and essential elements in human placenta have been summarized (e.g., [29,30]). Since Cd is the most present toxic metal in tobacco smoke, a comparative analysis of Cd in the placenta vs. other specimens showed that placental Cd levels are 10-fold to 100-fold higher than in maternal blood/serum and umbilical cord blood/serum, respectively. This also confirms that the placenta presents an effective barrier against direct fetal Cd toxicity in utero. The majority of reports to date refer to the placental Cd levels in tobacco smokers vs. nonsmokers [29]. We previously reported that placental Cd may serve as an additional valuable biomarker of active cigarette smoking that could be used in risk assessment of smoking in women during the childbearing period, especially in cases when maternal hair samples are lacking, of an insufficient length, or chemically treated, and are therefore inadequate for measuring nicotine content [59].

Essential microelements and cigarette smoke exposure. Maternal smoking may disturb the transplacental transport of trace elements to the fetus, such as elevated Fe and Cu, as found by other authors [60], and placental Zn, as reported in our previous study [40] in smokers. In this study, we found decreased placental Fe as reported earlier [33]; the levels of placental Zn, Cu, and Se were unchanged, whereas both urine cotinine and placental Cd and Zn were positively correlated. It was reported that smoking affects iron homeostasis mainly by changing hemoglobin concentrations, which are generally increased [61].

### 4.2. Cigarette Smoking, Trace Elements, and Steroid Hormones during Pregnancy

Active cigarette smoking was reported to reduce P4 and E2 in maternal serum during the third trimester [62], P4 in term placenta [33,63], and serum steroid hormones in nonpregnant smokers at the follicular stage of their menstrual cycle [64], suggesting increased smoking-related risks for reproductive disorders, preterm birth, and low birth weight. A long-term follow-up study spanning two decades showed an inverse relationship between maternal smoking during pregnancy and the age of their daughters’ menarche [65], and an increased ratio between free testosterone and free E2 in their sons [66]. After appropriate preparation of the human placental tissue (discussed in [7]), steroid hormones synthesized in the placenta can be analyzed and serve as biomarkers of reproductive EDC effects in situ, while androgen and estrogen concentrations measured in cord blood/serum represent steroidogenesis of the entire maternal–placental–fetal unit [67]. Our previous studies found cigarette smoking-related declines of placental P4 [33] and leptin levels [40], and provided evidence for the potential of Cd, as a tobacco smoke constituent, to act as an endocrine disruptor of reproduction in exposed women and in female rats [34]. A recent study on pregnant women from Puerto Rico measured Pb blood levels at two windows of rapid fetal growth (at weeks 18 ± 2 and 26 ± 2 of pregnancy), and found that they were associated with small increases in P4 concentrations [68]. In the present study, placental P4 and E2 levels were comparable with values reported previously [40], trending towards lower P4 and higher E2 in active smokers, and no effects on measured sex steroids were found in maternal serum.

In this study, maternal serum P4 and placental weight were positively correlated, regardless of maternal smoking. These results corroborate findings of positive associations between maternal levels of P4, estriol (E3), and testosterone with placental weight, while there was tendency of inverse association between cord blood steroids, significant only for testosterone, and placental weight [69]. Another study reported positive associations of maternal P4 and E3 with birth weight, more pronounced among taller mothers, while cord blood P4 and birth weight associations were inverse [70]. Our results, showing a positive correlation between maternal P4 and birth weight, along with a tendency of positive association between maternal P4 and birth length, are in line with those findings. We also found a positive correlation between birth weight and birth length and, contrary to the results by Lagiou et al. [70], we found a positive association between cord P4 and birth size, which was statistically significant between cord P4 and birth length. Additionally, our results showed an inverse association between placental E2 and birth size, significant for birth length. Diversity in the associations between described endpoints could be attributed to heterogeneity across the participants recruited in different countries, several decades apart in the studies with different designs, and using different methods for hormone analyses, to mention some of the possible reasons. However, our and other authors’ data show that placental weight and birth size variables, including birth weight and birth length, are associated with maternal and fetal sex steroid levels, which should be considered in studies investigating the possible relationship of endocrine factors with risk for health disorders in adulthood. Furthermore, we found higher levels of P4 in cord serum than in the maternal serum 10-fold in never smokers, and about 7-fold in active smokers, which corroborated the results of both above reported papers [69,70].

Irrespective of maternal smoking habit, levels of steroid hormones in cord serum of boys (*n* = 94) vs. girls (*n* = 61) did not differ; the measured means (IQR) were 805 (596–1089) ng/mL vs. 705 (547–1080) ng/mL for P4, and 10,875 (8107–14,925) pg/mL vs. 9559 (6407–13,510) pg/mL for E2. The results related to E2 are in line with findings of a large population-based study that evaluated umbilical cord estrogens and their relationship with perinatal characteristics [71]. In the cited study, it was reported that cord estrogens did not differ between male and female neonates, and twinning was consistently associated with lower E2 levels.

Only a few studies have examined the relationship between maternal tobacco smoking and steroid hormones in cord blood/serum. To date, reductions in E3, irrespective of neonatal sex, have been reported [72], as well as the findings indicating higher levels of testosterone in male, and higher levels of dehydroepiandrosterone (7α-OH-DHEA) in female newborns of smokers [73]. In our study, we found significantly lower levels of both measured cord serum steroid hormones, P4, and E2 in active smokers (Figure 1), which is to our knowledge, the first such results reported in the literature.

### 4.3. Strengths and Limitations of This Study

The main strengths of this study are the following. In addition to maternal blood/serum, we used noninvasive biological samples of urine, as well as human placenta and umbilical cord blood. The latter specimens have still been underrepresented in human biomedical studies, despite having the potential to provide valuable biomarkers of exposure and effects in environmental epidemiology. Placenta has been recognized as a dual valuable biological sample to assess both maternal and prenatal exposure. Similarly, cord blood collected at birth, as it reflects fetal circulation in late gestation, has the potential to measure concentrations of various xenobiotics, including toxic metal(loid)s and essential elements, in the maternal–placental–fetal unit. Moreover, analysis of the hormones in cord blood/serum, such as androgens and estrogens, is valuable in studies of perinatal effects, and as predictive biomarkers of future health disorders (discussed in [67]). Referring to the latter, steroid levels near the time of birth are influenced by myriad perinatal factors that should be considered when examining associations between cord steroid levels and biological endpoints, including gestational age, delivery mode, and type of pregnancy. We defined these endpoints among the inclusion criteria of our study to avoid side effects, apart from cigarette smoking as a source of toxic metals Cd and Pb exposure on steroids in the maternal–placental–fetal unit, which we also consider as a strength of this study.

Studies using the DOHaD approach are continuously and rapidly developing further by reviewing how fetal nutritional disruption during intrauterine development could be linked to different birth phenotypes, and how adaptive responses in utero may cause changes in placental and fetal hormone concentrations, and later, different metabolic abnormalities, chronic diseases, and malignant tumors in adulthood. We considered as a strength that our study aimed to follow that trial. Nevertheless, this study was conducted on a modest number of study participants, both in total (*n* = 155) and per each of three study groups, which we consider its major weakness. Additional limitations of this study lie in the fact that participants in the study groups designated as never smokers and former smokers were older and with higher education levels compared to active smokers, which contributed to the heterogeneity of the study groups. These confounders were not critical compared to the significant effects of maternal cigarette smoking on the observed parameters in this study.

## 5. Conclusions

This study provides additional data on toxic Cd and Pb, and essential element Fe interaction in the maternal–placental–fetal unit, and new data related to disruption of sex steroids in umbilical cord blood at term due to hazardous maternal cigarette smoking exposure. The results suggest that umbilical cord sex steroid levels could potentially serve as an early marker of developmental origins of individual burden of disease in the future, which deserves further study.

## Figures and Tables

**Table 1:** General characteristics of mother–newborn pairs (*n* = 155) in relation to maternal smoking.

	Never Smokers (*n* = 71)	Former Smokers (*n* = 48)	Active Smokers (*n* = 36)	*p*
Maternal characteristics				
Age (years)	33 (21–44) ^a^	33 (24–42) ^a^	29 (21–38) ^b^	**0.007**
Education				**0.0002**
Primary school	3 (4.2%)	2 (4.2%)	5 (14%)	
Secondary school	25 (35%)	21 (44%)	25 (69%)	
University degree	43 (61%)	25 (52%)	6 (17%)	
Prepregnancy BMI (kg/m^2^)	24 ± 3.9 23 (16–36)	24 ± 3.3 23 (18–31)	23 ± 4.5 23 (15–37)	0.874
BMI ≤ 18.5/18.5–24.9/≥ 25 (*n*)	2/48/21	2/30/16	5/19/12	
Weight gain during pregnancy (kg)	14 ± 4.5 13 (4.0–24)	15 ± 6.3 14 (5.0–38)	15 ± 5.3 14 (6.0–30)	0.763
Parity	2 (1–6)	2 (1–4)	2 (1–6)	0.097
Smoking habit ^†^				
Started smoking (age, years)	/	21 (15–32) ^a^	18 (11–27) ^b^	**0.005**
Smoking duration (years)	/	8 (2–21)	10 (2–20)	0.073
Smoking index ^1^	/	86 (28–202)	98 (2–235)	0.733
Passive smoking (*n*)	39	23	25	0.081
Cotinine in urine (µg/g creatinine)	1.6 (0.1–22) ^a^	4.9 (0.1–502) ^a^	1315 (2.6–5660) ^b^	**<0.001**
Newborn characteristics				
All newborns				
Birth weight (g)	3602 ± 504	3603 ± 511	3410 ± 433	0.159
Birth length (cm)	51 ± 1.8	51 ± 2.2	51 ± 1.9	0.471
Trimmed placental weight (g)	409 ± 90	397 ± 89	408 ± 76	0.493
Birth weight:placental weight ratio	9.1 ± 1.8 ^a,b^	9.3 ± 1.3 ^a^	8.5 ± 1.3 ^b^	**0.015**
Boys/Girls, *n*	46/25	25/23	23/13	0.356
Girls				
Birth weight (g)	3413 ± 502	3476 ± 379	3344 ± 392	0.444
Birth length (cm)	50 ± 1.9	50 ± 1.9	50 ± 1.4	0.839
Trimmed placental weight (g)	405 ± 90	385 ± 65	402 ± 63	0.635
Birth weight:placental weight ratio	8.6 ± 1.3	9.2 ± 1.1	8.4 ± 0.93	0.084
Boys				
Birth weight (g)	3704 ± 480	3720 ± 591	3448 ± 459	0.148
Birth length (cm)	51 ± 1.7	51 ± 2.4	50 ± 2.2	0.298
Trimmed placental weight (g)	411 ± 92	408 ± 106	411 ± 84	0.826
Birth weight:placental weight ratio	9.3 ± 1.9	9.4 ± 1.5	8.6 ± 1.5	0.095

Results are presented as mean ± SD, median (min-max) or number and percent (%). Differences between groups were tested by the Kruskal–Wallis test, and Dunn’s multiple comparisons test with Bonferroni adjustment was used as a post hoc test. Fisher’s exact test was used for categorical variables. Significant *p*-values (*p* < 0.05) are marked in bold. Values with different superscript letters in the same row are significantly different. BMI—body mass index. ^†^ Differences between former and active smokers tested by Mann–Whitney’s test. ^1^ Calculated as the number of cigarettes smoked per day *x* years of smoking.

**Table 2:** Concentration of Cd and Pb in mother–newborn pairs in relation to maternal smoking.

	Never Smokers (*n* = 71)	Former Smokers (*n* = 48)	Active Smokers (*n* = 36)	*p*
Cadmium (Cd)				
Maternal urine (µg/g creatinine)	0.25 ± 0.12 0.22 (0.15–0.32) ^a^	0.31 ± 0.19 0.25 (0.19–0.37) ^a,b^	0.34 ± 0.18 0.32 (0.23–0.40) ^b^	**0.004**
Maternal blood (µg/L)	0.28 ± 0.13 0.25 (0.19–0.34) ^a^	0.35 ± 0.18 0.31 (0.21–0.47) ^a^	1.3 ± 1.6 0.68 (0.52–1.2) ^b^	**<0.001**
Placenta (µg/kg)	6.0 ± 2.5 5.4 (4.3–7.7) ^a^	7.3 ± 3.2 6.6 (4.8–9.2) ^a,b^	9.0 ± 4.3 7.9 (5.6–12) ^b^	**0.001**
Cord blood (µg/L)	0.02 ± 0.01 0.02 (0.01–0.03) ^a^	0.02 ± 0.01 0.02 (0.01–0.03) ^a^	0.03 ± 0.01 0.03 (0.02–0.04) ^b^	**<0.001**
Lead (Pb)				
Maternal urine (µg/g creatinine)	0.57 ± 0.24 0.53 (0.38–0.73)	0.59 ± 0.31 0.56 (0.36–0.74)	0.66 ± 0.30 0.65 (0.45–0.78)	0.226
Maternal blood (µg/L)	9.3 ± 4.5 7.9 (6.1–11)	8.9 ± 4.1 8.7 (5.9–11)	10 ± 3.5 9.3 (8.0–11)	0.133
Placenta (µg/kg)	2.2 ± 2.0 1.6 (1.1–2.5) ^a^	2.5 ± 1.8 2.1 (1.4–3.0) ^a,b^	3.1 ± 2.1 2.5 (1.8–3.5) ^b^	**0.002**
Cord blood (µg/L)	6.1 ± 2.7 5.8 (4.1–7.2)	6.4 ± 3.4 5.8 (4.3–8.2)	6.8 ± 2.4 6.6 (5.2–8.1)	0.164

Results are presented as mean ± SD and median (25–75% interquartile range). Differences between groups were tested by Kruskal–Wallis test, and Dunn’s multiple comparisons test with Bonferroni adjustment was used as a post hoc test. Significant *p*-values (*p* < 0.05) are marked in bold. Values with different superscript letters in the same row are significantly different.

**Table 3:** Concentration of essential elements in mother–newborn pairs in relation to maternal smoking.

	Never Smokers (*n* = 71)	Former Smokers (*n* = 48)	Active Smokers (*n* = 36)	*p*
Iron (Fe)				
Maternal urine (mg/g creatinine)	0.03 ± 0.03 0.02 (0.01–0.03)	0.03 ± 0.03 0.03 (0.02–0.04)	0.02 ± 0.02 0.02 (0.01–0.02)	0.061
Maternal blood (mg/L)	443 ± 60 458 (410–480)	428 ± 70 445 (391–474)	433 ± 48 437 (404–467)	0.324
Placenta (mg/kg)	104 ± 29 99 (79–122) ^a^	111 ± 34 108 (86–129) ^a^	89 ± 27 85 (65–109) ^b^	**0.004**
Cord blood (mg/L)	575 ± 64 574 (532–613)	580 ± 74 583 (548–640)	561 ± 53 567 (524–592)	0.173
Zinc (Zn)				
Maternal urine (mg/g creatinine)	0.46 ± 0.24 0.44 (0.27–0.58)	0.47 ± 0.23 0.45 (0.31–0.55)	0.58 ± 0.36 0.47 (0.32–0.78)	0.372
Maternal blood (mg/L)	6.2 ± 0.84 6.4 (5.7–6.7)	6.0 ± 0.95 6.0 (5.5–6.5)	6.0 ± 0.73 6.1 (5.7–6.6)	0.112
Placenta (mg/kg)	11.5 ± 1.25 11 (11–12)	11.5 ± 1.3 11 (11–12)	12 ± 1.6 12 (11–13)	0.098
Cord blood (mg/L)	2.0 ± 0.38 2.0 (1.7–2.2)	2.0 ± 0.40 2.0 (1.7–2.3)	1.9 ± 0.53 1.8 (1.6–2.1)	0.230
Copper (Cu)				
Maternal urine (mg/g creatinine)	0.02 ± 0.01 0.02 (0.01–0.02)	0.02 ± 0.01 0.02 (0.02–0.02)	0.02 ± 0.01 0.02 (0.01–0.02)	0.082
Maternal blood (mg/L)	1.6 ± 0.29 1.6 (1.4–1.8)	1.6 ± 0.28 1.6 (1.5–1.7)	1.6 ± 0.24 1.6 (1.5–1.7)	0.958
Placenta (mg/kg)	1.2 ± 0.14 1.0 (0.96–1.1)	1.05 ± 0.115 1.0 (0.96–1.1)	1.1 ± 0.13 1.1 (0.98–1.2)	0.744
Cord blood (mg/L)	0.60 ± 0.07 0.60 (0.55–0.65)	0.595 ± 0.073 0.60 (0.57–0.64)	0.60 ± 0.08 0.59 (0.56–0.63)	0.88
Selenium (Se)				
Maternal urine (mg/g creatinine)	0.03 ± 0.01 0.03 (0.02–0.03) ^a^	0.03 ± 0.02 0.03 (0.02–0.03) ^a^	0.02 ± 0.01 0.02 (0.02–0.03) ^b^	**0.019**
Maternal blood (mg/L)	0.10 ± 0.02 0.10 (0.09–0.11)	0.10 ± 0.02 0.10 (0.09–0.11)	0.09 ± 0.01 0.09 (0.09–0.10)	0.061
Placenta (mg/kg)	0.17 ± 0.02 0.17 (0.16–0.18)	0.17 ± 0.02 0.17 (0.16–0.18)	0.16 ± 0.02 0.16 (0.15–0.17)	0.344
Cord blood (mg/L)	0.10 ± 0.02 0.09 (0.09–0.11)	0.10 ± 0.02 0.10 (0.09–0.11)	0.09 ± 0.01 0.09 (0.09–0.10)	0.276

Results are presented as mean ± SD and median (25–75% interquartile range, IQR). Differences between groups were tested by Kruskal–Wallis test, and Dunn’s multiple comparisons test with Bonferroni adjustment was used as a post hoc test. Significant *p*-values (*p* < 0.05) are marked in bold. Values with different superscript letters in the same row are significantly different.

**Table 4:** Results of multiple linear regression analyses for dependent variables that were significantly associated with active smoking in both univariate and multivariate analyses ^a^.

	P4 Cord Serum	E2 Cord Serum	Cd Maternal Urine	Cd Maternal Blood	Cd Placenta	Cd Cord Blood	Pb Placenta	Fe Placenta
Intercept	6.6 [6.0, 7.3] *p* < **0.001**	9.5 [8.8, 10] *p* < **0.001**	−3.1 [−3.8, −2.4] *p* < **0.001**	−1.8 [−2.7, −0.94] *p* < **0.001**	0.81 [0.14, 1.5] *p* = **0.017**	−4.1 [−5.2, −2.9] *p* < **0.001**	0.07 [−1.0, 1.2] *p* = 0.905	4.4 [4.0, 4.9] *p* < **0.001**
Smoking ^b^								
Active smokers	−0.19 [−0.38, −0.004] *p* = **0.045**	−0.29 [−0.49, −0.08] *p =* **0.006**	0.32 [0.12, 0.52] *p* = **0.002**	1.1 [0.85, 1.4] *p* < **0.001**	0.37 [0.17, 0.56] *p* < **0.001**	0.66 [0.34, 0.98] *p* < **0.001**	0.44 [0.12, 0.76] *p* = **0.007**	−0.13 [−0.27, −0.003] *p* = **0.045**
Former smokers	−0.07 [−0.23, 0.09] *p* = 0.377	−0.13 [−0.31, 0.04] *p* = 0.128	0.17 [−0.01, 0.34] *p* = 0.059	0.20 [−0.03, 0.42] *p* = 0.085	0.13 [−0.04, 0.29] *p* = 0.133	−0.10 [−0.37, 0.18] *p* = 0.495	0.14 [−0.13, 0.41] *p* = 0.316	0.03 [−0.08, 0.14] *p* = 0.613
Passive smoking	−0.02 [−0.16, 0.12] *p* = 0.797	0.02 [−0.14, 0.17] *p* = 0.822	−0.003 [−0.16, 0.15] *p* = 0.974	0.21 [0.01, 0.41] *p* = **0.036**	0.13 [−0.01, 0.28] *p* = 0.077	−0.17 [−0.41, 0.08] *p* = 0.180	0.07 [−0.18, 0.31] *p* = 0.590	−0.002 [−0.10, 0.10] *p* = 0.974
Age	0.001 [−0.02, 0.02] *p* = 0.905	−0.01 [−0.03, 0.01] *p* = 0.492	0.04 [0.03, 0.06] *p* < **0.001**	0.01 [−0.01, 0.04] *p* = 0.283	0.03 [0.02, 0.05] *p* < **0.001**	0.02 [−0.01, 0.05] *p* = 0.250	0.02 [−0.01, 0.05] *p* = 0.201	0.01 [−0.002, 0.02] *p* = 0.096
University degree	0.05 [−0.11, 0.20] *p* = 0.565	0.01 [−0.16, 0.19] *p* = 0.874	−0.17 [−0.34, −0.002] *p* = **0.047**	−0.12 [−0.34, 0.10] *p* = 0.294	−0.09 [−0.25, 0.08] *p* = 0.293	−0.04 [−0.32, 0.23] *p* = 0.747	−0.11 [−0.38, 0.16] *p* = 0.414	−0.05 [−0.16, 0.06] *p* = 0.413
Prepregnancy BMI	0.01 [−0.01, 0.02] *p* = 0.486	0.01 [−0.01, 0.03] *p* = 0.297	0.01 [−0.01, 0.03] *p* = 0.573	−0.01 [−0.04, 0.01] *p* = 0.315	−0.01 [−0.03, 0.01] *p* = 0.156	−0.01 [−0.05, 0.02] *p* = 0.359	−0.02 [−0.05, 0.01] *p* = 0.211	0.003 [−0.01, 0.02] *p* = 0.653
Weight gain	0.02 [0.01, 0.03] *p* = **0.004**	0.01 [−0.01, 0.02] *p* = 0.429	0.01 [−0.003, 0.02] *p* = 0.132	0.01 [−0.01, 0.03] *p* = 0.262	0.01 [−0.003, 0.02] *p* = 0.112	−0.02 [−0.04, 0.01] *p* = 0.171	0.03 [0.004, 0.05] *p* = **0.020**	−0.0002 [−0.01, 0.01] *p* = 0.961
Parity	−0.15 [−0.23, −0.08] *p* < **0.001**	−0.12 [−0.21, −0.04] *p* = **0.003**	0.02 [−0.06, 0.10] *p* = 0.655	0.07 [−0.03, 0.18] *p* = 0.188	−0.03 [−0.10, 0.05] *p* = 0.511	−0.03 [−0.16, 0.10] *p* = 0.658	−0.04 [−0.17, 0.09] *p* = 0.514	−0.09 [−0.14, −0.04] *p* = **0.001**
Adjusted R^2^	0.22	0.14	0.22	0.44	0.17	0.12	0.08	0.11

^a^ Only models for dependent variables that were significantly associated with active smoking in both univariate and multivariate analyses are shown. For each independent variable in the model, regression coefficient with 95% confidence interval and *p*-value are shown. Significant *p*-values (*p* < 0.05) are marked in bold. All dependent variables were log-transformed. ^b^ Reference category: never smokers.

**Table 5:** Spearman’s correlation coefficients (*⍴*) for relationships between concentrations of progesterone (P4), estradiol (E2), and newborn data.

	Placental Weight	Birth Weight	Birth Length	Birth Weight:Placental Weight Ratio
Maternal serum P4	**0.22**	**0.23**	0.13	−0.10
Placental P4	−0.01	0.11	0.09	0.09
Cord serum P4	0.06	0.16	**0.16**	0.06
Maternal serum E2	0.04	0.04	0.05	−0.01
Placental E2	−0.06	−0.12	**−0.18**	−0.03
Cord serum E2	−0.10	−0.01	0.06	0.14

Significant correlations (at *p* < 0.05) are marked in bold.

## Data Availability

The datasets generated during and/or analyzed during the current study are available from the corresponding author on reasonable request.
